# Transcriptome analysis of embryonic domains in Norway spruce reveals potential regulators of suspensor cell death

**DOI:** 10.1371/journal.pone.0192945

**Published:** 2018-03-02

**Authors:** Salim H. Reza, Nicolas Delhomme, Nathaniel R. Street, Prashanth Ramachandran, Kerstin Dalman, Ove Nilsson, Elena A. Minina, Peter V. Bozhkov

**Affiliations:** 1 Department of Plant Biology, Uppsala BioCenter, Linnean Center for Plant Biology, Swedish University of Agricultural Sciences, Uppsala, SE, Sweden; 2 Department of Molecular Sciences, Uppsala BioCenter, Linnean Center for Plant Biology, Swedish University of Agricultural Sciences, Uppsala, SE, Sweden; 3 Umeå Plant Science Centre, Department of Forest Genetics and Plant Physiology, Swedish University of Agricultural Sciences, Umeå, Sweden; 4 Umeå Plant Science Centre, Department of Plant Physiology, Umeå University, Umeå, Sweden; 5 Department of Organismal Biology, Uppsala BioCenter, Linnean Center for Plant Biology, Uppsala University, Uppsala, SE, Sweden; Wuhan University, CHINA

## Abstract

The terminal differentiation and elimination of the embryo-suspensor is the earliest manifestation of programmed cell death (PCD) during plant ontogenesis. Molecular regulation of suspensor PCD remains poorly understood. Norway spruce (*Picea abies*) embryos provide a powerful model for studying embryo development because of their large size, sequenced genome, and the possibility to obtain a large number of embryos at a specific developmental stage through somatic embryogenesis. Here, we have carried out global gene expression analysis of the Norway spruce embryo-suspensor *versus* embryonal mass (a gymnosperm analogue of embryo proper) using RNA sequencing. We have identified that suspensors have enhanced expression of the NAC domain-containing transcription factors, *XND1* and *ANAC075*, previously shown to be involved in the initiation of developmental PCD in *Arabidiopsis*. The analysis has also revealed enhanced expression of Norway spruce homologues of the known executioners of both developmental and stress-induced cell deaths, such as metacaspase 9 (MC9), cysteine endopeptidase-1 (CEP1) and ribonuclease 3 (RNS3). Interestingly, a spruce homologue of bax inhibitor-1 (*PaBI-1*, for *Picea abies BI-1*), an evolutionarily conserved cell death suppressor, was likewise up-regulated in the embryo-suspensor. Since *Arabidopsis BI-1* so far has been implicated only in the endoplasmic reticulum (ER)-stress induced cell death, we investigated its role in embryogenesis and suspensor PCD using RNA interference (RNAi). We have found that PaBI-1-deficient lines formed a large number of abnormal embryos with suppressed suspensor elongation and disturbed polarity. Cytochemical staining of suspensor cells has revealed that PaBI-1 deficiency suppresses vacuolar cell death and induces necrotic type of cell death previously shown to compromise embryo development. This study demonstrates that a large number of cell-death components are conserved between angiosperms and gymnosperms and establishes a new role for BI-1 in the progression of vacuolar cell death.

## Introduction

Plant embryogenesis starts with the asymmetric division of the zygote in the plane perpendicular to the future apical-basal axis of the embryo. This division generates a small apical cell and a large basal cell, the progenitors of two structurally and functionally distinct domains: embryo proper (in angiosperms) or embryonal mass (EM, in gymnosperms) and suspensor, respectively [1]. The apical domain gives rise to the plant, whereas the suspensor functions as a conduit of growth factors and nutrients to the growing apical domain and is gradually eliminated through programmed cell death (PCD).

The terminal differentiation and elimination of the embryo-suspensor is the earliest manifestation of PCD in plant life. In Norway spruce (*Picea abies* L. Karst.), the suspensor contains several files of elongated cells, derived through a series of asymmetric cell divisions in the EM. Once produced, these cells undergo terminal differentiation and embark on the PCD pathway. Generation of new layers of suspensor cells thus results in a gradient of PCD stages along apical-basal axis of an embryo. While suspensor cells adjacent to the EM are at the commitment stage of PCD, the cells at the lower layers of the suspensor are characterized by increased degree of dismantling. Therefore, position of the cell within the suspensor of spruce embryos can be used as a marker of PCD stage [2, 3, 4].

Most examples of plant developmental PCD, including the death of the embryo-suspensor, belong to the class of vacuolar cell death [5]. During vacuolar cell death, the cell contents are removed completely by a combination of autophagy-like engulfment of the cytoplasm and organelles and vacuolar collapse. Necrosis is another major class of plant PCD characterized by mitochondrial dysfunction and early rupture of plasma membrane, resulting in incomplete removal of cell contents [5]. It has been shown that genetic suppression of vacuolar PCD in the terminally-differentiated cells can trigger necrosis [6].

Our understanding of the molecular machinery regulating developmental PCD in plants is advancing, yet remains limited compared to animal-specific apoptosis. During terminal differentiation, the plant cell achieves the competency for death through expression of transcription factors (TFs) that regulate expression of genes controlling PCD triggers and executioner [7, 8]. Ethylene, reactive oxygen species (ROS), calcium influx and a decrease in pH have all been implicated as potential PCD triggers [7, 9].

Autophagy and activity of hydrolytic enzymes, such as cysteine, serine and aspartic proteases and nucleases execute PCD and are directly responsible for cell dismantling and morphology of cell corpse. In *Arabidopsis*, a type II metacaspase AtMC9, XYLEM CYSTEINE PEPTIDASES 1 and 2 (XCP1 and XCP2) are involved in post mortem clearance of root xylem cell contents [10]. In Norway spruce, execution of vacuolar PCD in the embryo-suspensor requires activity of a type II metacaspase mcII-Pa and autophagy [4, 6, 11]. A S1-P1 type nuclease BIFUNCTIONAL NUCLEASE 1 (BFN1) participates in DNA degradation during lateral root cap cell death [12].

The large size of Norway spruce embryos, which contain several millimetre-long suspensors (as compared to the *Arabidopsis* suspensor being composed of a single file of 6–9 small cells), the use of somatic embryogenesis to provide an unlimited number of genetically identical embryos at a specific developmental stage and the sequenced genome make somatic embryos of Norway spruce a powerful model system for studying molecular mechanisms of developmental PCD. Here, we took advantage of this system to compare transcriptomes of the living (EM) and dying (embryo-suspensor) domains of plant embryos using high-throughput RNA sequencing (RNA-Seq). Our analysis revealed a subset of genes highly expressed in the suspensor and therefore representing potential PCD initiators and executioners. Among these genes, we have found a spruce homologue of *BAX INHIBITOR 1* (*BI-1*), which has previously been shown to act as a suppressor of ER stress-mediated cell death in *Arabidopsis* [13]. Silencing of Norway spruce *BI-1* (*PaBI-1*, for *P*. *abies BI-1*) by RNA interference (RNAi) induced a switch from vacuolar to necrotic cell death in the suspensor leading to abnormal embryo development. Our findings thus not only define that an anti-necrotic role of BI-1 is conserved between angiosperms and gymnosperms but also connect this role to the regulation of developmental PCD.

## Materials and methods

### Norway spruce somatic embryo culture

Two embryogenic cell lines (88:22 and 11:18) originated from zygotic embryos of two independent fertilization events were cultured as previously described [14]. Briefly, the lines were maintained by weekly subculture on half-strength solidified LP medium containing growth regulators 9.0 μM 2,4-diclorophenoxyacetic acid (2,4-D) and 4.4 μM 6-benzylaminopurine (6-BA) (hereafter referred as proliferation medium). To stimulate embryo development, the cell lines were transferred onto solidified half-strength LP medium without growth regulators for one week followed by an additional week on solidified embryo maturation medium DKM supplemented with 30 μM abscisic acid (ABA). The cultures were grown in the dark at 22^o^ C.

Individual somatic embryos during the transition from early embryogeny to late embryogeny were split into EMs and suspensors under a Zeiss STEMI SV8 stereoscope (Germany). Briefly, the EM was hold in a petri plate with fine forceps and dissected by cutting through the first layer of the suspensor using a Feather surgical blade (Japan). The separated embryonic domains (S1 Fig) were treated with RNA stabilization reagent RNAlater (Qiagen) in a screwcap tube and then snap-frozen in liquid nitrogen before storage at -80^o^.

### RNA extraction, cDNA synthesis and RNA-seq

Total RNA was extracted from the EMs and suspensors using RNAqueous Micro kit (Ambion). RNA quality was assessed in terms of RNA integrity number (RIN) by Bioanalyzer (Agilent 2100 expert). To obtain sufficient amount of RNA required for RNA-seq, the RNA extracted from the embryos of cell line 11:18 was subsequently amplified using MessageAmpIIaRNA Kit (Ambion).

cDNA library preparation and subsequent sequencing were performed at the SciLifeLab (Stockholm, Sweden). Strand-specific cDNA libraries were prepared with TruSeq Stranded mRNA Sample prep kit of 96 dual indexes (Illumina, CA, USA) according to the manufacturer’s instructions except for the following changes. The protocols were automated in Agilent NGS workstation (Agilent, CA, USA) using purification steps as previously described [15, 16]. Clonal clusters were generated using cBot (Illumina) and sequenced on HiSeq2500 (Illumina) according to manufacturer's instructions. Bcl to Fastq conversion was performed with bcl2Fastq v1.8.3 from the CASAVA software suite. The quality scale was Sanger / phred33 / Illumina 1.9. The obtained data were deposited to the European Nucleotide Archive (ENA) and is accessible under the accession number PRJEB22154.

### Pre-processing of RNA-seq data and differential expression analyses

The data pre-processing was performed as described at http://www.epigenesys.eu/en/protocols/bio-informatics/1283-guidelines-for-rna-seq-data. Briefly, the quality of the raw sequence data was assessed using FastQC (http://www.bioinformatics.babraham.ac.uk/projects/fastqc/). Residual ribosomal RNA (rRNA) contamination was assessed and filtered using SortMeRNA (v1.8; [17]; settings—log—paired_in) using the rRNA sequences provided with SortMeRNA (rfam-5s-database-id98.fasta, rfam-5.8s-database-id98.fasta, silva-bac-16s-database-id85.fasta, silva-euk-18s-database-id95.fasta, silva-bac-23s-database-id98.fasta and silva-euk-28s-database-id98.fasta). Data were then filtered to remove adapters and trimmed for quality using Trimmomatic (v0.32; [18]; settings TruSeq3-PE-2.fa:2:30:10 SLIDINGWINDOW:5:20 MINLEN:50). After both filtering steps, FastQC was run again to ensure that no technical artefacts were introduced. Filtered reads were aligned to v1.0 of the Norway spruce genome (retrieved from the ConGenIE resource; [19]) using STAR (v2.3.1; [20]; non-default settings:—runThreadN 16—readFilesCommand zcat—outReadsUnmapped Fastx—outSAMstrandField intronMotif–alignIntronMax 100000). The annotations obtained from the Norway spruce v1.0 GFF file contain only one transcript per gene-model, which was used for gene level expression quantification. This GFF file and the STAR read alignments were used as input to the HTSeq [21] htseq-count python utility to calculate exon-based read count values. The htseq-count utility takes only uniquely mapping reads into account.

Statistical analysis of single-gene differential expression between EM and suspensor was performed in R (v3.3.2; R Core Team 2015) using the Bioconductor (v3.4; [22]) DESeq2 package (v1.14.1; [23]). FDR adjusted p-values were used to assess significance; a common FDR threshold of 1% was used throughout. For the data quality assessment (QA) and visualization, the read counts were normalized using a variance stabilizing transformation (VST) as implemented in DESeq2. The biological relevance of the data *e*.*g*. biological replicates similarity was assessed by Principal Component Analysis (PCA) and other visualizations (*e*.*g*. heatmaps), using custom R scripts. An overview of the data, including raw and post-QC read counts and alignment rates is given in S1 Table.

### Annotation of differentially expressed genes (DEGs) and gene ontology (GO) enrichment analysis

Fasta sequences for the DEGs were extracted from ConGenIE.org [19] and imported to Blast2GO for Gene Ontology (GO) enrichment analyses. For gene annotation, Basic Local Alignment Search Tool (http://www.ncbi.nlm.nih.gov/BLAST) of Blast2GO Version 4 [24] was used to find sequences similar to the DEGs. GO mapping option of Blast2GO was used to retrieve GO terms associated to the hits obtained by the BLAST search. For functional enrichment analysis, the numbers of annotated sequences in each GO term were counted by Blast2GO. Categorization of TFs was done by comparing the DEGs with the *P*. *abies* TFs listed in the Plant Transcription Factor Database (PlantTFDB) version 4.0 [25].

### Quantitative real-time PCR (qRT-PCR)

cDNA was synthesized from 500 μg of RNA isolated from embryogenic cell line 11:18 using Maxima First Strand cDNA synthesis kit (Thermo Scientific). A twentieth part concentration of each cDNA sample was utilized for the analysis using Dynamo Flash SYBR Green kit (Thermo Scientific) in a CFX PCR thermal cycler (sequences of all primers used in this study are listed in S2 Table). ΔΔC_T_ method was used to measure the fold expression of five genes of interest normalized to the expression of two reference genes: *cell division control 2* (*CDC2*) and *phosphoglucomutase* which were selected on the basis of their stability tested by Biogazelle-qbase+ software.

### Cloning of *PaBI-1* hairpin construct

For creating the hairpin of Norway spruce *PaBI-1* the corresponding parts of *PaBI-1* cDNA were amplified using attB1_AS_BI1_F/AsBI1_R_HindIII and attB2_S_BI1_R/S_BI1_F_HindIII using Phusion DNA polymerase (Thermo Scientific) and cut with *HindIII* (Thermo scientific), which produced 360 bp and 466 bp fragments of *PaBI-1* coding DNA sequences, respectively. The fragments were ligated with T4 DNA Ligase (Thermo Scientific) and recombined into pDONR/Zeocin (Zeo) vector by BP Clonase (Invitrogen) followed by recombination into modified pMDC32 vector containing suspensor-specific NIP (nodulin-like intrinsic protein) promoter [26] by LR Clonase (Invitrogen). The resulting recombined vector was checked by digestion with restriction enzymes followed by sequencing.

### Transformation

To suppress *PaBI-1* expression with the hairpin construct, *Agrobacterium*-mediated transformation of embryogenic cell lines 11:18 was performed as described previously [27]. pMDC32::PaBI-1 was transformed into *Agrobacterium tumefaciens* strain GV3101. Overnight culture of transformed *Agrobacterium* was centrifuged and resuspended in infiltration buffer (10 mM MES, 10 mM MgCl_2_, pH 5.5) to an OD_600_ of 10. Resuspended bacteria were mixed with 5-day-old spruce proliferating culture in 1:9 ratio. Acetosyringone was added to the mixed culture to a final concentration of 150 μM and the culture was left for 5 h with regular shaking. Thereafter the culture was transferred onto a filter paper placed on solidified proliferation medium and left for 2 days at 22°C in the dark after which, the filter paper was transferred to a fresh proliferation medium supplemented with 400 μg/ml Timentin and 250 μg/ml cefotaxime and cultured for additional 5 days under the same conditions. Next, the cells on filter paper were transferred to proliferation medium with 5 μg/ml hygromycin, 400 μg/ml Timentin and 250 μg/ml cefotaxime and incubated for 7 days under the same condition. At the next and each following subculture with 7-day intervals, the concentration of hygromycin was increased to 7.5 μg/ml, while other components remained the same. Once transformants emerged, they were transferred to a fresh proliferation medium with increasing concentration of hygromycin (10 μg/ml and 15 μg/ml) every week and finally maintained on the proliferation medium with 15 μg/ml hygromycin.

### Embryo staining and confocal microscopy

To detect the presence of dead cells, 10 ml of 5-day-old spruce culture resuspended in liquid proliferation medium devoid of growth regulators was stained with 0.0025% (w/v) Evans blue solution for 10 min at room temperature as described previously [6]. An aliquot of stained embryos was transferred onto a glass slide and imaged with a bright field microscopes (Zeiss Axioplan) with 2.5X Plan-Neofluar objective, NA 0.075. Images were taken using a DFC295 camera and LAS AF v3.2 software. The length of the suspensor was measured with ImageJ software.

For detection of necrotic cells, 5-day-old embryogenic suspension culture grown in liquid medium without growth regulators was stained first with 2 μg/ml FDA for 15 min and then counterstained with 1 μM FM4-64 for maximum 10 min as described previously [6]. The cells were imaged within 10 min after staining with FM4-64 using the sequential mode of a Zeiss LSM 780 confocal microscope, with a 20X objective, NA 0.8, excitation at 488 nm and 561 nm and emission at 490–587 nm and 582–754 nm for detection of FDA and FM4-64 staining, respectively.

## Results and discussion

### Overview of Norway spruce somatic embryo transcriptome

**Fig 1:**
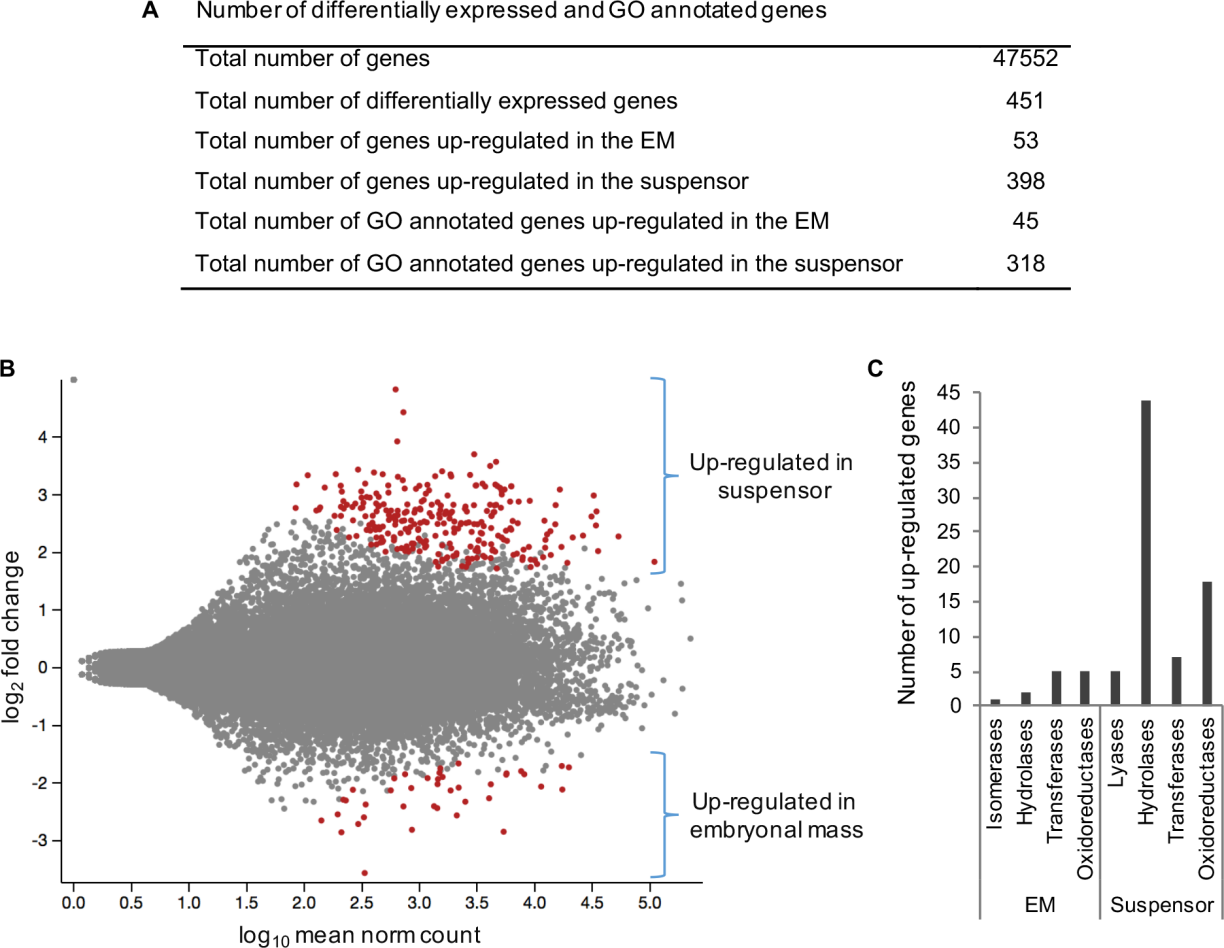
Summary of differentially-expressed genes in the Norway spruce EM versus suspensor. **(A)** Number of differentially expressed and GO annotated genes. **(B)** Glimma plot of expression values of RNA-seq detected genes normalized by their sequencing depth. Highlighted red are groups of genes up-regulated in the suspensor or EM. **(C)** Classes of transcriptionally up-regulated enzymes in the EM and in the suspensor.

To identify genes differentially expressed in the EM *versus* suspensor, RNA was isolated from the corresponding domains of somatic embryos originated from two unrelated genotypes (*viz*. cell lines). Thus, four RNA-Seq libraries were sequenced using Illumina technology which generated a total of ~410 million raw reads, the expression proxy of 47,552 genes (Fig 1A and 1B). Expression of 32.8% percent (23,184) of the 70,736 Norway spruce genes was not detected in the embryos. Accounting for the difference in library size (*i*.*e*. sequencing depth) and the technical and biological variability of the samples, a total of 451 genes were found to be differentially expressed between the two embryonic domains at a 1% adjusted p-value cutoff (False Discovery Rate). Of these 451 genes, 53 and 398 were up-regulated in the EM and suspensor, respectively (S1 and S2 Datasets; Fig 1A and 1B).

### Genes up-regulated in the EM

**Fig 2:**
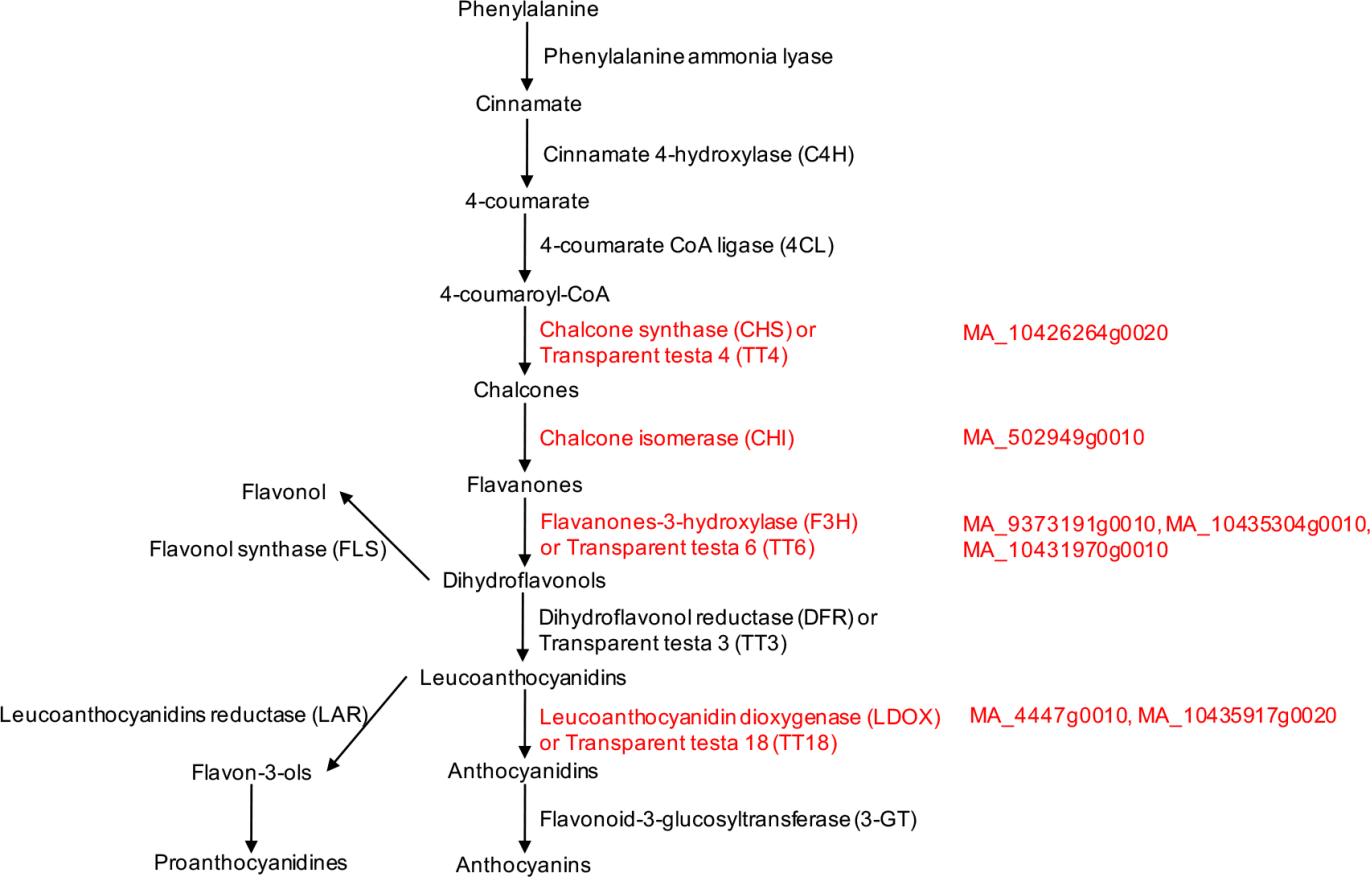
Schematic overview of flavonoid biosynthesis pathway (modified after [30]). Highlighted in red are enzymes showing transcriptional up-regulation in the EM and their corresponding ConGenIE ID numbers.

About 85% (45 out of 53) of the genes up-regulated in the EM could be mapped to GO (Fig 1A; S1 Dataset). Using Blast2GO PRO analysis we have identified that a significant fraction of these genes (13 out of 45) encode enzymes possessing oxidoreductase, transferase, hydrolase or isomerase activity (Fig 1C, Table 1). Of these enzymes, seven fall into flavonoid biosynthesis pathway [28] (Fig 2), suggesting enhanced ROS scavenging activity maintained in the meristematic cells of the EM [29].

**Table 1:** Examples of enzymes transcriptionally up-regulated in the EM of Norway spruce and their known functions.

	Enzyme class	ConGenIE ID	Log_2_ fold change	Arabidopsis homologue	Function	References
EM	Isomerase	MA_502949g0010	2.11	Chalcone-flavanone isomerase	Flavonoid biosynthesis	[30]
	Hydrolases	MA_10426205g0010	2.54	β-Glucosidase	Hydrolysis of β 1,4-glucosidic bonds, plant pathogen defence	[31]
		MA_4342g0010	2.25	Phospholipase A2	PIN protein trafficking to the plasma membrane, Repression of the AtMYB30 to negatively regulate HR and defense reponses	[32, 33]
	Transferase	MA_10426249g0010	1.91	Glutathione-S-transferase	Detoxification of xenobiotics	[34]
		MA_5735g0010	1.83	Chalcone synthase (TT4)	Flavonoid biosynthesis	[30]
	Oxidoreductase	MA_194138g0010	2.71	Alcohol dehydrogenase	Glycolysis, fatty acid degradation. Resistance to anoxia or hypoxia, cold and osmotic stresses	[35]
		MA_4447g0010	2.03	Anthocyanidin synthase (TT18)	Anthocyanidin biosynthesis	[30]
		MA_10433569g0010	2.11	Anthocyanidin reductase	Proanthocyanidin biosynthesis	[36]

Using the Plant Transcription Factor Database [25] we identified six MYB and MYB-like TFs up-regulated in the EM (Table 2). The function of most of these TFs in *Arabidopsis* was linked to the regulation of flavonoid biosynthesis [37, 38]. In particular, MYB12 was shown to activate promoters of several flavonoid biosynthesis enzymes, such as chalcone synthase (TT4), flavanone 3-hydroxylase (TT6), flavonol synthase (FLS) and chalcone flavanone isomerase (CHI) [39]. In agreement, TT4, TT6 and CHI were transcriptionally upregulted in the EM (Fig 2; S2 Fig).

**Table 2:** Transcription factors up-regulated in the EM of Norway spruce and functions of their Arabidopsis homologues.

ConGenIE ID	Log_2_ fold change	Arabidopsis homologue	Function	References
MA_8147g0020,	2.84	AtMYB3	Repressor of phenylpropanoid biosynthesis gene expression	[37]
MA_130918g0010,	2.32			
MA_8626g0010	2.85			
MA_9991g0010	2.59	R3-type MYB TF	Regulator of epidermal cell differentiation	[38]
MA_21440g0010	2.13	AtMYB12	Activator of flavonoid biosynthesis pathway enzymes	[39]
MA_14452g0010	2.09	AtMYB7	Repressor of flavonol biosynthesis	[40]

Polar auxin transport is essential for the correct apical-basal patterning of conifer embryos [41]. Flavonoids modulate the activity of auxin-transporting P-glycoproteins and may also be involved in modulating the activity of regulatory proteins *e*.*g*. phosphatases and kinases [42]. Thus, up-regulation of flavonoid biosynthesis genes and related TFs may promote spruce embryo development by regulating polar auxin transport and functions of housekeeping enzymes, in addition to more general role of flavonoids in ROS scavenging and abiotic stress resistance.

### Genes up-regulated in the embryo-suspensor

Altogether, about 80% (318 out of 398) of the genes up-regulated in the suspensor were assigned to a GO category (Fig 1A; S2 Dataset), functional enrichment analysis of which revealed that a significant fraction of these genes (74 out of 318) encode enzymes possessing oxidoreductase, transferase, hydrolase or lyase activity, with hydrolases being clearly predominating (Fig 1C; Table 1).

#### Genes related to cell elongation and cell wall modification

The suspensor cells in Norway spruce achieve highly-elongated morphology accompanied by growth of lytic vacuoles prior to tonoplast collapse and complete clearance of cellular contents [1]. This developmental process requires abundance of structural materials (*e*.*g*. carbohydrates and lipids), as well as activity of cell wall modifying enzymes. We have found transcriptional up-regulation of aquaporins, which accelerate water uptake to facilitate cell expansion [43] and choline kinase (CK) (as detected by qRT-PCR, S2 Fig), which is involved in the biosynthesis of phosphatidylcholine [44], the major component of plasma membrane and tonoplast [45]. Enzymes participating in cell wall loosening and reorganization, such as xyloglucan endotransglucosylase/hydrolase, galactosidases and pectinesterase were likewise up-regulated in the embryo-suspensor (Table 3).

**Table 3:** Cell wall modifying enzymes transcriptionally up-regulated in the Norway spruce embryo-suspensor and functions of their Arabidopsis homologues.

ConGenIE ID	Log_2_ fold change	Arabidopsis homologue	Function	References
MA_96657g001,	2.17	Xyloglucan endotransglucosylase /hydrolase	Breaking down cellulose-xyloglucan matrix during cell expansion, fruit ripening, fruit softening and senescence	[46, 47]
MA_11177g0010	2.07			
MA_54403g0020	2.56	α-Galactosidase	Removal of galactosyl residues from pectin and xyloglucan	[48]
MA_217488g0010	2.40	β-Galactosidase		
MA_87592g0010	3.09	Pectinesterase 11	Methyl esterification of carboxyl groups (COO^-^) of pectin polysaccharide homogalacturonan to reduce their negative charge and blocking Ca^2+^ crosslinking during cell wall expansion and ripening of fruits	[48, 49]

#### Transcription factors

In animals, besides a major role of post-translational regulation, developmental apoptosis is also controlled at the transcriptional level [50]. Our analysis of the transcriptome of the Norway spruce embryo-suspensor suggests that transcriptional control may also be a part of the regulatory network responsible for initiation and execution of developmental PCD *in planta*. We identified nine TFs with enhanced expression in the suspensor. These genes belong to six families of TFs, including bHLH (basic Helix-Loop-Helix), C2H2 (Cys2His2-like fold containing), ERF (ETS2 repressor factor), LBD (Lateral organ boundary domain), MYB (Myeloblastosis) and NAC [for NAM (no apical meristem), ATAF (Arabidopsis transcription activation factor), CUC (cup-shaped cotyledon)]. *Arabidopsis* homologues of these genes were previously implicated in the regulation of a plethora of PCD-dependent developmental processes, including reproductive organ development, embryogenesis, vascular differentiation and senescence (Table 4).

**Table 4:** Transcription factors up-regulated in the Norway spruce embryo-suspensor and the functions of their Arabidopsis homologues.

ConGenIE ID	Log_2_ fold change	TF family	Arabidopsis homologue	Function	References
MA_97786g0010	1.99	bHLH	ZHOUPI	Embryonic cuticle development	[51]
MA_70076g0010	2.42	C2H2	AT5G03510.1	Salinity, heat and osmotic stress tolerance	[52]
MA_203191g0010	1.97	ERF	ATERF-9	Biotic stress resistance (*e*.*g*., necrotrophic fungi)	[53]
MA_328535g0010	2.40	LBD	LBD11	Unknown	
MA_10431212g0010	2.47	MYB	MYB26	Anther dehiscence	[54]
MA_115536g0010	1.99	MYB-related	LHY	Circadian stress-induced expression of cell death marker genes.	[55]
MA_18153g0010	2.65	NAC	NAC025	Embryogenesis and degeneration of ovule integuments	[56]
			XND1	Tracheary element differentiation	[56]
MA_402393g0010	2.81	NAC	ANAC075	Secondary cell wall formation and xylem vessel differentiation	[57]
MA_75192g0010	2.35	NAC	ANAC72	Leaf senescence and dehydration response	[58]

#### Cell-death triggers and stress-responsive genes

Reactive oxygen species (ROS) act as signalling molecules to control plant developmental PCD [7]. We observed enhanced expression of three homologs of L-ascorbate oxidase in the Norway spruce embryo-suspensor (Table 5). It has been shown that enhanced expression of L-ascorbate oxidase causes accumulation of H_2_O_2_ and changes redox homeostasis [59]. A gene encoding another H_2_O_2_ producing enzyme, germin, which is known to act at early stages of stress-induced cell death [60], was likewise up-regulated in the suspensor (Table 5). Germin has previously been shown to be transcriptionally up-regulated in the suspensor of *Larix marschlinsii* somatic embryos, where it has been suggested to also participate in cell wall remodelling [61].

**Table 5:** Examples of potential anti- and pro-cell-death genes up-regulated in the Norway spruce embryo-suspensor and functions of their Arabidopsis homologues.

ConGenIE ID	Log2 fold change	Arabidopsis homologue	Function	References
MA_629271g0010,	3.01	Aquaporin	Increased water uptake to facilitate cell expansion	[43]
MA_68132g0010,	2.71			
MA_9571426g0010,	2.73			
MA_9821440g0010,	2.86			
MA_18297g0010,	2.1			
MA_3515726g0010,	2.0			
MA_5484215g0010	2.82			
MA_10236360g0010,	3.14	L-ascorbate oxidase	Production of H_2_O_2,_ cell wall remodelling	[59]
MA_6574321g0010,	3.0			
MA_9371g0010	2.76			
MA_828526g0010	2.35	Germin	Production of H_2_O_2_, cell wall remodelling	[66]
MA_103463g0010	3.57	CEP1	Tapetal PCD and pollen development	[67]
MA_616703g0010	3.11	AtMC9	Post-mortem xylem vessel clearance	[10]
MA_117445g0010,	2.51	Cathepsin B-like cysteine protease	HR-, UV-, oxidative- and ER stress-associated PCD	[68–70]
MA_8902970g0010	2.8			
MA_4032367g0010	2.74	RNS3	Senescence	[71]
MA_405561g0010	2.16	Cytochrome p450	Osmotic stress response, hypersensitive response (HR) to bacteria and senescence	[63, 66]
MA_242814g0010	1.9	Alcohol oxidase	Anoxia tolerance	[64]
MA_10427493g0010,	3.18	Heat shock proteins (HSPs)	Protein stabilization and refolding	[65, 72]
MA_10427493g0030	2.49			
MA_43661g0010	2.57	AtBI-1	Cell-death suppressor	[13, 73]
MA_10425833g0010	2.38	AtBAG-1	Proteasomal degradation of misfolded protein	[65]

Apart from the potential cell-death triggers, we have also found increased expression of a number of genes associated with stress response (Table 5). These include a cytochrome p450 involved in plant response to osmotic stress [62], hypersensitive response (HR) to bacteria and senescence [63]; alcohol oxidase involved in anoxia tolerance through alcoholic fermentation [64] and two genes encoding heat shock proteins (HSPs). Interestingly, we also found enhanced expression of a homologue of evolutionary conserved ER-stress induced cell-death suppressor *BAX INHIBITOR 1* (*BI-1*), *PaBI-1* (Table 5; S2 Fig) [13] and *Bcl2-associated athanogene 1* (*BAG1*) implicated in proteasomal degradation of misfolded protein [65].

#### Proteases and nucleases

Among hydrolytic enzymes transcriptionally up-regulated in the embryo-suspensor, several cysteine peptidases have previously been reported to be involved in PCD and development of different plant tissues and organs [10, 11, 67] (Table 5). For example, the Norway spruce gene MA_103463g0010 is a homologue of *Arabidopsis* papain-like cysteine protease CEP1, which mediates tapetal PCD and pollen development [67], whereas gene MA_616703g0010 is a homologue of *Arabidopsis* METACASPASE 9 (*AtMc9*) participating in post-mortem xylem vessel clearance [10]. The *Arabidopsis* homologues of two up-regulated genes for cathepsin B-like cysteine protease were previously implicated in senescence, HR, UV, oxidative stress- and ER stress-mediated PCD [68–70].

Noteworthy, RNA-Seq analysis did not reveal transcriptional up-regulation of another type-II metacaspase, *mcII-Pa*, which is known to play a major role in PCD of Norway spruce embryo-suspensor [4,11]. Changes of *mcII-Pa* gene are too subtle for us to observe them, or the changes in that gene are not common to all individual cells within a pool and hence the effect is diluted or neutralised—*i*.*e*. unobservable at that resolution. Post-translational modification of mcII-Pa, *i*.*e*., autoprocessing and calcium binding, required to convert *mcII-Pa* zymogen to active enzyme [11] could be another explanation of this finding.

Besides protease encoding genes, we have found enhanced expression of *Arabidopsis RIBONUCLEASE 3* (*RNS3*) homologue (Table 5). In *Arabidopsis*, *RNS3* expression is elevated during senescence [71]. RNS3 is conserved across plant and animal kingdoms [74, 75] and its animal homologue RNase T2 has been reported to control melanocyte apoptosis [75].

### *PaBI-1* is required for embryo patterning and suppression of necrotic cell death

BI-1 is an evolutionary conserved cell-death suppressor localized to the ER membrane and regulating calcium and lipid dynamics under ER stress [13, 76]. Although *BI-1* has been implicated to suppress chemically induced ER stress-mediated cell death, as well as cell death induced by necrotrophic fungi and heat stress [13, 73], the function of *BI-1* in plant developmental PCD remains unknown.

**Fig 3:**
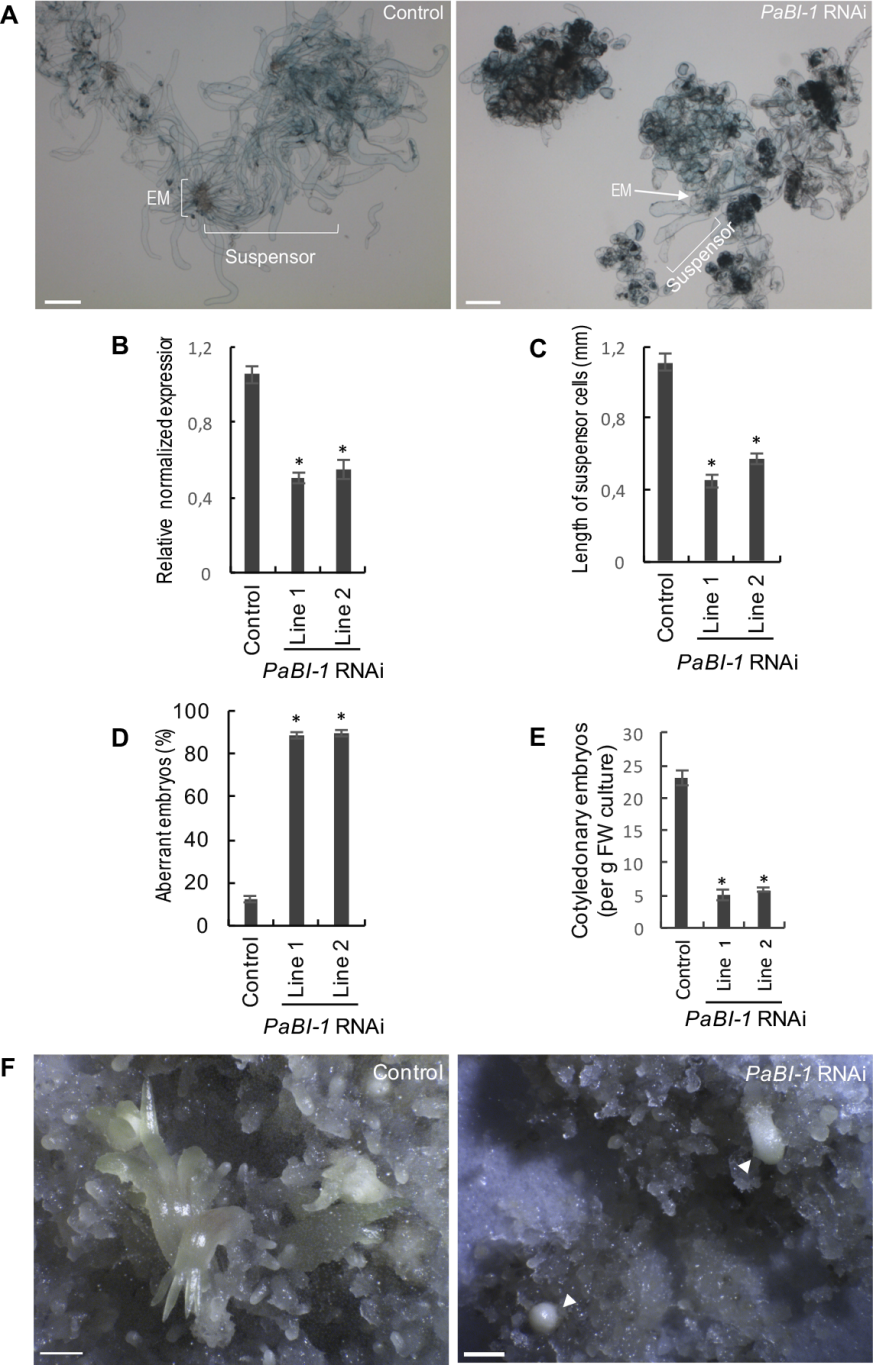
PaBI-1 deficiency impairs embryogenesis. (**A**) Morphology of control (transformed with pMDC32::GUS, [6]) and *PaBI-1* RNAi lines grown for 5 days without growth regulators and stained with Evan’s blue to detect dying or dead cells. Scale bars, 500 μm. (**B**) Normalized expression of *PaBI-1* in the control and RNAi lines. *, P<0.01; *vs* control, Student’s *t*-test. (**C**) Length of suspensor cells. Data represent mean ± SEM for more than 80 Evan’s blue-positive suspensor cells from at least 10 different embryos per line. *, P<0.0001; *vs* control, Student’s *t*-test. **(D)** Frequency of aberrant early embryos lacking elongated suspensor. Data represent mean ± SEM. The experiment included more than 40 embryos per line and was repeated two times. *, P<0.0001; *vs* control, Student’s *t*-test. **(E)** Number of cotyledonary embryos formed after 7 weeks on ABA-containing medium. Data represent mean ± SEM from three independent experiments, each including one plate per line. *, P<0.001; *vs* control, Student’s *t*-test. FW, fresh weight. **(F)** Morphology of maturing embryos in control and *PaBI-1* RNAi lines grown for 9 weeks on ABA-containing medium. Arrowheads indicate under-developed embryos in *PaBI-1* RNAi line, compared to fully developed embryos that have already started germinating in the control line. Scale bars, 2 mm.

To investigate the role of PaBI-1 in embryo development and associated PCD, we suppressed *PaBI-1* expression using RNAi (Fig 3A and 3B). The gene silencing impaired apical-basal patterning (Fig 3A) through suppression of anisotropic expansion of the suspensor cells, as revealed by measuring the length of Evan’s blue positive cells (Fig 3C). This accounted for the increased frequency of aberrant early embryos lacking properly formed suspensors in the *PaBI-1* RNAi lines (Fig 3D) and the decreased number of cotyledonary embryos (Fig 3E and 3F).

**Fig 4:**
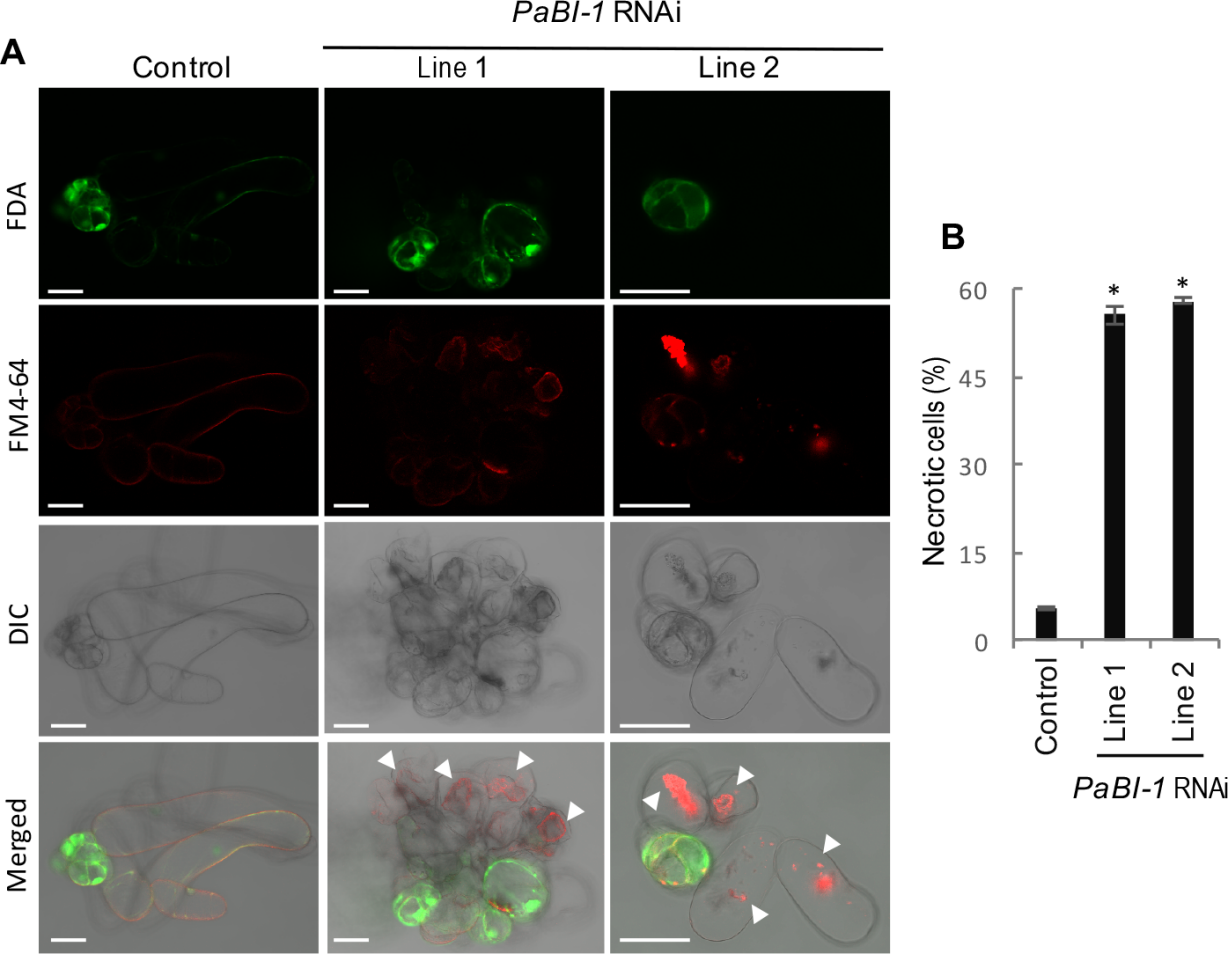
PaBI-1 deficiency induces necrotic cell death. (**A**) FDA and FM4-64 staining of control and *PaBI-1* RNAi lines to detect necrosis. Arrowheads indicate shrunken and undigested protoplast in necrotic cells. DIC, differential interference contrast. Scale bars, 100 μm. (**B**) Frequency of necrotic cells. Data represents mean ± SEM from three independent experiments, each including more than 50 cells per line. (*, P<0.0001; *vs* control, Student’s *t*-test).

Based on the morphological criteria, most examples of plant cell death can be divided in two main classes: vacuolar cell death and necrosis [5]. Silencing of *PaBI-1* switched the mode of cell death in the suspensor from vacuolar to necrotic, as indicated by the presence of shrunken and largely undigested protoplast (Fig 4A). Quantification of this necrotic hallmark using double staining with FDA and FM4-64 has shown ten-fold increase in the frequency of necrotic cells in *PaBI-1* RNAi lines, as compared with the control line (Fig 4B).

In animals, BI-1 functions as a suppressor of apoptosis induced by cell-death effector Bax. Plants lack direct homologues of Bax and other members of Bcl-2 family, but surprisingly, a homologue of BI-1 is present in plants [77]. The mechanism by which PaBI-1 suppresses necrosis and sustains progression of vacuolar cell death in spruce embryo-suspensor remains to be identified. Yet, one can propose that this mechanism is reliant on either maintenance of ER homeostasis or interaction with autophagy pathway or a combination of both for the following two reasons.

Firstly, ER stress is induced by accumulation of misfolded or unfolded proteins. In *Arabidopsis*, BI-1 has been shown to be transcriptionally up-regulated upon ER stress-induced unfolded protein response (UPR). AtBI-1 keeps the cell alive until the ER homeostasis is re-established by the activity of ER chaperons such as *Bip2* [13]. The enhanced expression of *PaBI-1* in the suspensor suggests that this developmental PCD may likewise implicate ER stress. Vacuolar cell death is believed to be a slow process [5] and the cell remains metabolically active until vacuolar collapse. We speculate that down-regulation of *PaBI*-*1* compromises ER homeostasis in the terminally-differentiated suspensor cells, thus triggering necrosis.

Secondly, it has been recently reported that in *Nicotiana benthamiana*, tobacco BI-1 interacts with autophagy-related protein ATG6 and silencing of *BI-1* reduced autophagic flux and enhanced *N*-mediated hypersensitive response (HR) cell death [78]. In spruce, autophagy sustains vacuolar cell death and prevents necrosis of the suspensor cells [6]. Therefore, PaBI-1 deficiency–induced necrotic death of the suspensor cells might be due to compromised autophagy, the possibility that calls for further investigations.

## Conclusions

**Fig 5:**
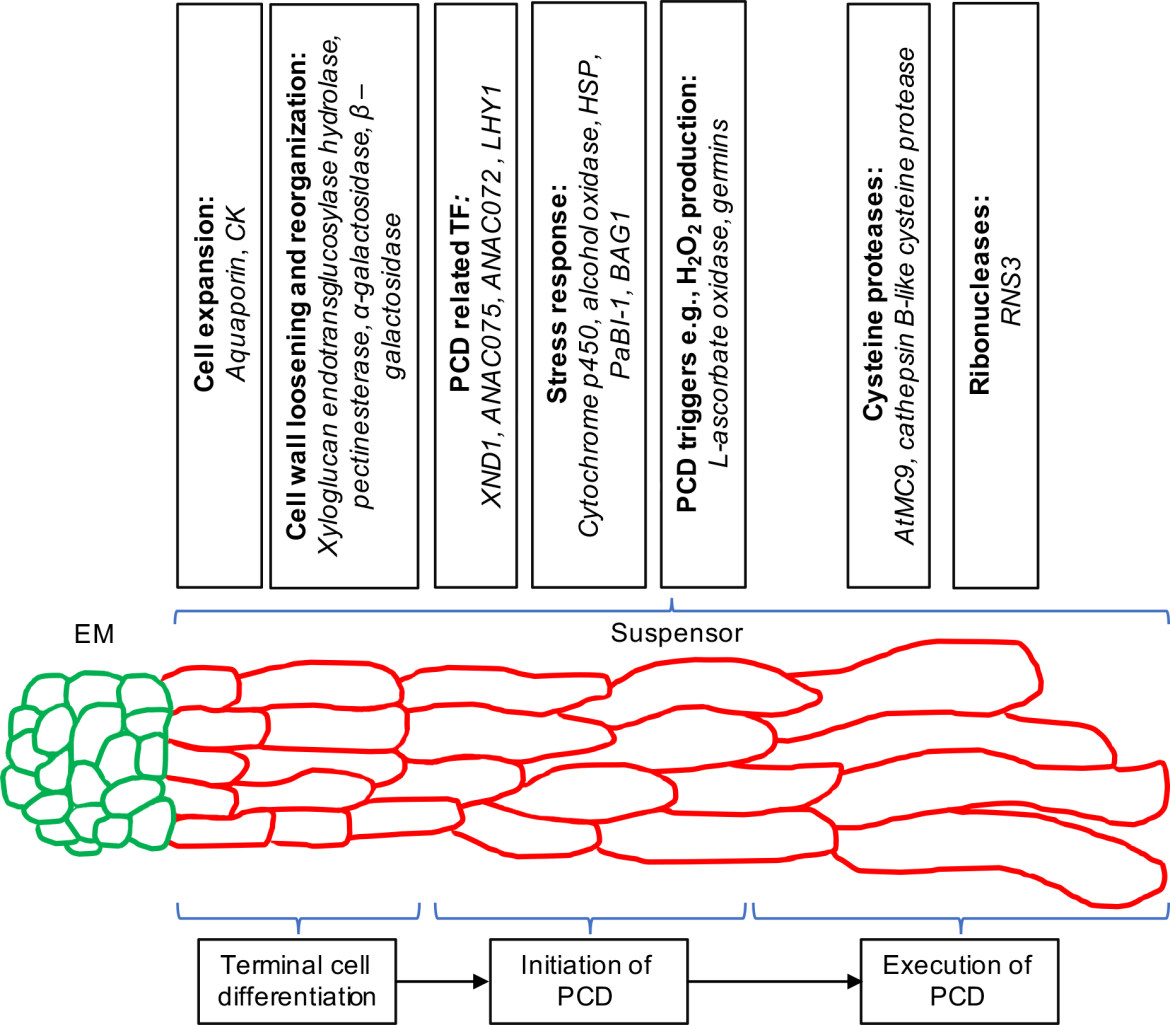
A hypothetical model of transcriptionally regulated processes and corresponding up-regulated genes involved in terminal differentiation and death of the Norway spruce embryo-suspensor, as suggested by RNA-seq analysis. Cells in the upper layers of the suspensor (i.e. adjacent to the embryonal mass) begin to expand with simultaneous reorganization of cell wall and enlargement of vacuole. Thereafter cells initiate PCD by expressing transcription factors and stress-responsive genes. The latter might act to suppress rapid cell death and to allow gradual cell dismantling characteristic for vacuolar PCD. At the basal end of the suspensor, hydrolytic enzymes (proteases and nucleases) execute PCD by processing protein and nucleic acid substrates. Note that some gene names correspond to *Arabidopsis* homologues.

In this study, we performed global transcriptome analysis of the two embryonic domains of Norway spruce to identify potential regulators of suspensor PCD. Since the embryo-suspensor in Norway spruce is composed of several cell layers representing a gradient of successive stages of PCD [1–3], we attempted to ascribe identified suspensor-specific DEGs to the sequence of events underlying terminal cell differentiation, initiation and execution of PCD (Fig 5). Our observations suggest that suspensor cells adjacent to the EM and undergoing terminal differentiation express genes controlling loosening and reorganization of the cell wall, as well as aquaporin and CK, thus facilitating cell expansion and vacuole enlargement. Next, the elongated suspensor cells initiate PCD by expressing a subset of dedicated TFs, which in turn confer simultaneous expression of PCD triggers (*e*.*g*. L-ascorbate oxidase and germins) and stress-response genes preventing rapid cell collapse (*e*.*g*. *PaBI-1*) and sustaining slow progression of vacuolar cell death. It is conceivable that all or some of these TFs, in particular *XND1* and *ANAC075*, are already highly expressed in the first suspensor cell layer composed of cells undergoing terminal cell differentiation to orchestrate expression of genes regulating cell wall loosening and reorganization. Finally, execution of PCD at the basal end of the suspensor requires enhanced expression of hydrolytic enzymes acting to degrade proteins and nucleic acids (Fig 5). Clearly, a higher resolution, single cell layer-specific mRNA isolation procedure assisted by laser capture microdissection is required to verify the proposed sequence of transcriptional changes occurring throughout Norway spruce embryo-suspensor.

Olvera-Carrillo and colleagues [74] have recently performed a comparative bioinformatics analysis of transcriptomes of developmentally-regulated cell deaths in *Arabidopsis* and concluded that various examples of developmental PCD share a common set of regulators, including RNS3, BFN1, PASPA3, AtMc9 and SCPL48. Availability of conifer genomes enables evolutionary insight into molecular regulation of plant development in gymnosperm *versus* angiosperm lineages, and our transcriptomics data suggest significant conservation of developmental cell death components between the lineages. This conservation encompasses both TFs (*e*.*g*. ANAC072, ANAC075 and XND1) and downstream effectors, such as RNS3 and metacaspase-9. Identification of anti-cell death protein BI-1 as a potential regulator of Norway spruce suspensor PCD indicates that there might yet be some interesting differences between angiosperms and gymnosperms. Further functional studies are required to link PaBI-1 and other candidate components of suspensor PCD into biochemical pathways.

## Supporting information

S1 Dataset List of genes up-regulated in Norway spruce embryonal mass. (XLSX)

S2 Dataset List of genes up-regulated in Norway spruce embryo suspensor. (XLSX)

S1 Table Overview of RNA-seq data. (XLSX)

S2 Table Primers used in this study. (DOCX)

S1 Fig DIC images of separated embryonal mass and suspensor. Norway spruce embryo during the transition from early to late embryogeny was dissected into embryonal mass (A) and suspensor (B). Red lines indicate the dissection plane where the embryogenic domains were separated. Scale bars 100 μm.
(TIF)

S2 Fig Quantitative real-time PCR analysis of selected DEGs. ΔΔC_T_ method was used to measure gene expression in the EM or suspensor, which was normalized to two reference genes *Cell division control 2* (*CDC2)* and *Phosphoglucomutase* (*PHOS*). *CK1*, *Choline Kinase 1; ENDO2*, *Endonuclease 2; TT4*, *Transparent testa 4; D6PK*, *D6 protein kinase; PaBI-1*, *Picea abies Bax inhibitor 1*. (*, P<0.0001; *vs* control, Student’s *t*-test).
(TIF)
