# Supplementary material for: Transcriptome analysis of embryonic domains in Norway spruce reveals potential regulators of suspensor cell death
Source: PLoS One. 2018 Mar 2;13(3):e0192945. doi: 10.1371/journal.pone.0192945 (PMC5834160; doi:10.1371/journal.pone.0192945)
Supplement: S2 Table — (DOCX) [file pone.0192945.s004.docx]

**S2 Table.** **Primers used in this study**

| **Primer** | **5’--> 3’ sequence** |
| --- | --- |
|  |  |
| **Primers for cloning** |  |
| attB1_AS_BI1_F | GGGGACAAGTTTGTACAAAAAAGCAGGCTGTGGAATAAATATCTGAATCAG |
| AsBI1_R_HindIII | AAAAGCTTCGATCGCCCATTTCATCG |
| attB2_S_BI1_R | GGGGACCACTTTGTACAAGAAAGCTGGGTGTGGAATAAATATCTGAATCAGAAT |
| S_BI1_F_HindIII | AAAAGCTTGATGAAATTTCGCTCCGG |
|  |  |
| **Primers for qRT-PCR** |  |
|  |  |
| PaCK1_qF | GTTCCTTCCCTACTGGTCGC |
| PaCK1_qR | TCCACAGCTTTGGTTGACGA |
| PaD6PK_qF | ACAGCCGGGCATGAGATTTA |
| PaD6PK_qR | TCTAAGGCCAGCAACACCTC |
| PaENDO2_qF | AAGAAGCATCGGAGGCAGT |
| PaENDO2_qR | TTATGGCACCTTCAAGGCA |
| PaBI-1_qF | TTGCACCCATTCTCTTCGCT |
| PaBI-1_qR | TATAATGGACGCCAGAGCCC |
| PaTT4_qF | TGTGCGACAAGTCGGCAATA |
| PaTT4_qR | CTCGCAGACGTTGGGATTCT |

F, forward; R, reverse.
