# Supplementary figures and images for: Transcriptome analysis of embryonic domains in Norway spruce reveals potential regulators of suspensor cell death
Source: PLoS One. 2018 Mar 2;13(3):e0192945. doi: 10.1371/journal.pone.0192945 (PMC5834160; doi:10.1371/journal.pone.0192945)

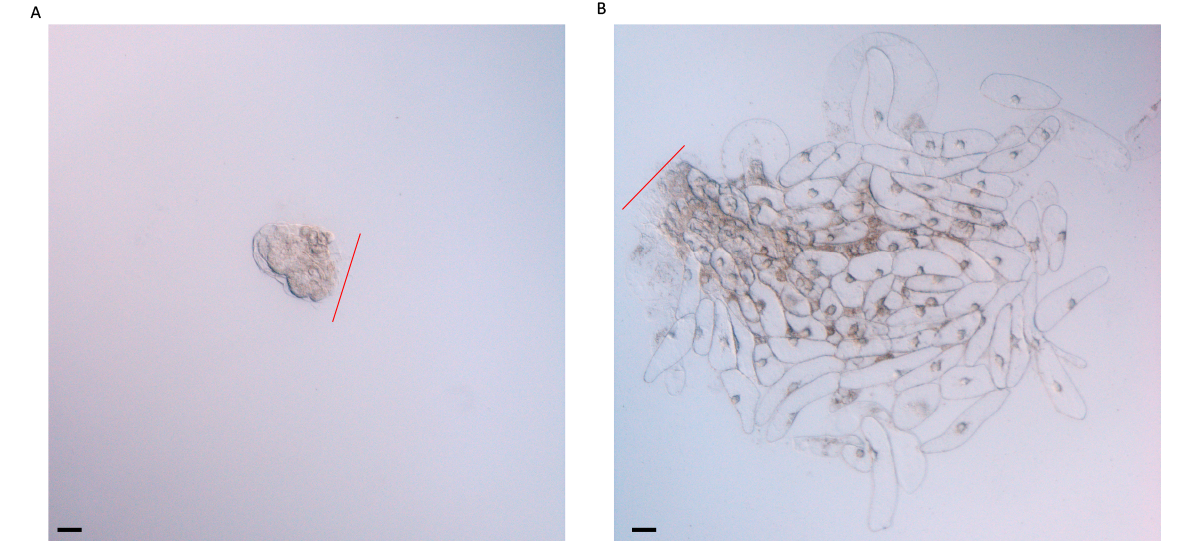

Supplement: S1 Fig — Norway spruce embryo during the transition from early to late embryogeny was dissected into embryonal mass (A) and suspensor (B). Red lines indicate the dissection plane where the embryogenic domains were separated. Scale bars 100 μm. (TIF) [file pone.0192945.s005.tif]

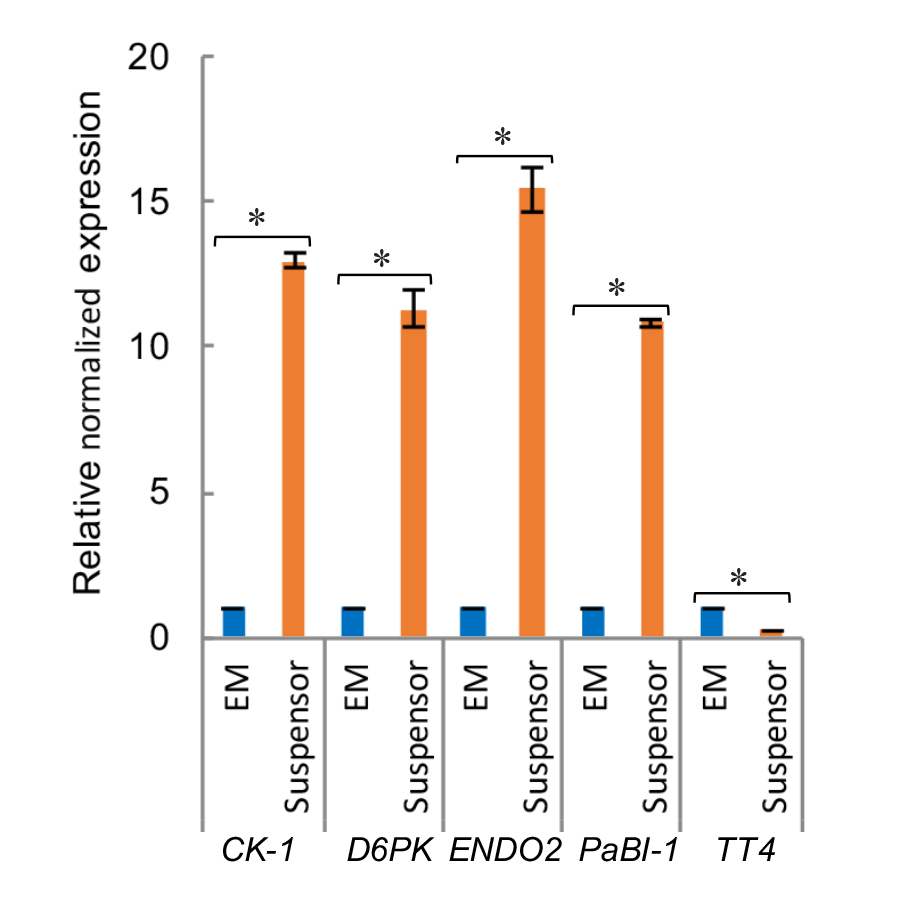

Supplement: S2 Fig — ΔΔCT method was used to measure gene expression in the EM or suspensor, which was normalized to two reference genes Cell division control 2 (CDC2) and Phosphoglucomutase (PHOS). CK1, Choline Kinase 1; ENDO2, Endonuclease 2; TT4, Transparent testa 4; D6PK, D6 protein kinase; PaBI-1, Picea abies Bax inhibitor 1. (*, P<0.0001; vs control, Student’s t-test). (TIF) [file pone.0192945.s006.tif]
